# Influence of Carbonyl Iron Particles (CIP) and Glass Microspheres on Thermal Properties of Poly(lactic acid) (PLA)

**DOI:** 10.3390/polym16152194

**Published:** 2024-08-01

**Authors:** Travis L. Thornell, Dane N. Wedgeworth, Mark D. Antwine, Jedadiah F. Burroughs

**Affiliations:** Geotechnical and Structures Laboratory, U.S. Army Engineer Research and Development Center, 3909 Halls Ferry Rd., Vicksburg, MS 39180, USA; dane.n.wedgeworth@usace.army.mil (D.N.W.); mark.d.antwine@usace.army.mil (M.D.A.); jedadiah.f.burroughs@usace.army.mil (J.F.B.)

**Keywords:** extrusion, thermal analysis, rheology, 3-D printing, metal particles, glass particles, polylactic acid

## Abstract

In this investigation, composite poly(lactic acid) (PLA) systems of hollow glass microspheres (MS) and carbonyl iron particles (CIP) were processed and characterized to investigate the effects of using conductive and insulating particles as additives in a polymer system. PLA-MS and PLA-CIP were set at the two levels of 3.94 and 7.77 vol.% for each particle type to study the effects of the particle material type and loading on neat PLA’s thermal properties. It was observed during the twin-screw extrusion that the addition of CIP greatly decreased the viscosity of the PLA melt during processing. Correlations determined using thermogravimetric analysis, differential scanning calorimetry, thermal conductivity, and shear rheology provided insights into how thermal stability was affected. The incorporation of MS and CIP altered thermal properties such as the glass transition temperature (T_g_), melting temperature (T_m_), and cold crystallization temperature (T_cc_). The metal CIP-filled systems had large increases in their thermal conductivity values and viscoelastic transitions compared to those with PLA that were correlated with the observed overheating during extrusion.

## 1. Introduction

With the growing popularity of additive manufacturing, e.g., 3-D printing, a renewed interest in advanced polymer nanocomposites and blends has arisen [1]. Polymer nanocomposites can be easily obtained via extrusion-based melt processing. Thermoplastic-based materials are combined with fillers to create nano- and microcomposites that exhibit a range of improved mechanical and thermal properties. Fused filament fabrication (FFF) processing techniques have greatly utilized thermoplastic composites to produce additively manufactured parts [2,3,4]. The FFF method uses a filament that is then extruded in the melt state, and, with the aid of computer software, printing paths are followed to build structures up layer by layer. The ability to control various printing parameters gives researchers the ability to understand the influence of many variables on the material properties. Rajpurohit and Dave investigated PLA prints by manipulating the raster height, raster width, and layer height while keeping the temperature and infill parameters constant [2].

Poly(lactic acid) (PLA) is one of the most well-known commercial biopolymers in use today, and PLA-based materials have been a popular choice in FFF processing. Its biocompatibility, recyclability, and ease of processing are the advantages of using PLA in manufacturing and 3-D printing [5,6]. Nanocomposites with PLA comprising many functional fillers such as graphene [7,8,9], carbon nanotubes [10,11], metals [12,13,14,15], cellulose nanocrystals [16,17,18], and other biopolymers [19,20] have been investigated. Laureto et al. studied PLA–metal composites containing copper, bronze, steel, and magnetic iron and correlated the obtained filler loadings with thermal conductivity but described limited correlations with other thermal properties [12]. The inclusion of advanced fillers has provided improvements to neat PLA’s mechanical and thermal properties. The effect of colorants and pigments has also been shown to affect material properties and processing conditions [5,21]. The uses of PLA and PLA-based nanocomposites in additive manufacturing and 3-D printing have increased demands on improving the strength and thermal stability of PLA-based materials. Notably, the glass transition temperature (T_g_) and melting temperature (T_m_) can be manipulated by the choice of the filler type and loading [22].

The production of PLA composites can be achieved with extrusion or melt blending, compression molding, and solution processing. For most processing routes, conditions (zone temperatures, pressure, print speed, etc.) are kept constant to isolate the effects of additives on the properties of the host material. For mixing processes that rely on temperature, care must be taken to not thermally degrade the material during heating. It becomes important to understand the effects of changing the thermal conductivity of the neat material and nanocomposites formed.

Other considerations of nanocomposites are the changes in the rheology and viscoelasticity of the host polymer [19,23]. The rheological profiles of PLA and PLA-based nanocomposites can be used to understand processability and how the fillers are dispersed within a matrix. The shape, density, chemistry, and loading level of fillers and additives have major impacts on the overall rheology of the system. In our previous efforts, we investigated the role that colorants played in the thermal properties of commercial PLA filaments [24] and the combined modeling and experimental effects that carbonyl iron particles (CIP) and ceramic glass microspheres (MS) have on PLA’s and poly(ethylene terephthalate glycol)’s (PETG’s) mechanical properties [25].

In this work, we report the effects of CIP, a thermally conductive additive, and MS, an insulating additive, when incorporated within a PLA matrix. The observed changes in the processing via twin-screw extrusion are coupled with thermal analyses and rheology to develop an understanding of how thermally conductive and insulating fillers alter a thermoplastic material. The dramatic effects of particle concentration, material composition, and density greatly influence the PLA composite materials’ thermal properties.

## 2. Materials and Methods

### 2.1. Materials

The primary polymeric material used in this study was Translucent Clear Pro Series PLA sourced from MatterHackers (Lake Forest, CA, USA). This material was obtained as a filament with a standard diameter of 1.75 mm and a reported density of 1.25 g/cm^3^. The filament was pelletized before extrusion to facilitate the incorporation of additives. Two additives were included to study the thermal effects of fillers on neat PLA: CIP and MS. CIP (CM grade) were sourced from BASF (Florham Park, NJ, USA), having a median particle size of 10 μm and a reported density of 7.9 g/cm^3^. G-800 ceramic microspheres were purchased from Zeeospheres Ceramic, LLC (Lockport, LA, USA), having a median particle size of 14 μm and a reported density of 2.2 g/cm^3^. The median particle sizes for both additives were determined using laser diffraction techniques on a solution of particles dispersed in deionized water. Composites were produced at 3.94% and 7.77% by bulk volume for each particle type. The bulk volume was used to standardize the additive rates to account for the hollow nature of the MS.

### 2.2. Methods

#### 2.2.1. Extrusion

Extrusion was performed using a Thermo Fisher Process 11 twin-screw extruder (Waltham, MA, USA). A calibration curve was calculated by collecting a minimum of three samples from both the additive and polymer volumetric feeders for one minute each to determine linearity. Using a linear fitting approach, the polymer and additive feed rates with the least deviation were selected to yield the desired composite weight percent. Based on the melting point of the neat PLA, the temperature profile chosen for this material was 200 °C for zones 2–8 across the barrel and 190 °C for the die, where zone one was water-chilled. This profile was chosen to ensure proper flow and low torque on the extruder. Once a steady-state flow was achieved, disks (2 mm thickness × 25 mm diameter) were injection-molded using a Thermo Scientific HAAKE MiniJet Pro Injection Molding system (Waltham, MA, USA) at 200 °C and 400 psi. The excess flash material on the disks was trimmed using a razor blade before evaluation.

#### 2.2.2. Density

The densities of the PLA, PLA-CIP, and PLA-MS samples were determined using Archimedes’ method using a Sartorius YDK01 Density Determination Kit (Goettingen, Germany) as described in ASTM D792.

#### 2.2.3. Thermogravimetric Analysis

Thermogravimetric analysis (TGA) was used to understand the loading of the additives and overall thermal stability. TGA was conducted using a TA Instruments TGA 5500 (New Castle, DE, USA). Samples were run in high-temperature platinum pans to 1000 °C at 10 °C/min under nitrogen atmosphere.

#### 2.2.4. Differential Scanning Calorimetry

Differential scanning calorimetry (DSC) analysis was performed on a TA Instruments DSC 250 (New Castle, DE, USA) using a heat–cool–heat sequence from 0 °C to 220 °C with heating rates of 10 °C/min and cooling rates of 5 °C/min. For modulated DSC (MDSC), samples were equilibrated to −90 °C before modulating the temperature by 1 °C for 60 s before ramping at 3 °C/min to 200 °C.

#### 2.2.5. Thermal Conductivity and Effusivity

Thermal conductivity measurements were obtained using a C-Therm Trident (Fredericton, NB, CAN) thermal conductivity instrument with the modified transient plane source (MTPS) method following ASTM D7984 [26] using water as contact fluid in a digitally controlled oven. The thermal conductivity was calculated through Equation (1):

(1) $$k\left(\frac{W}{m*K}\right)=\frac{\varepsilon^{2}}{\rho *C_{p}},$$

where k is the thermal conductivity in the units of watts per meter-Kelvin, ε is the effusivity, ρ is the density as measured using Archimedes’ method, and Cp is the specific heat from the DSC measurements.

#### 2.2.6. Shear Rheology

Injection-molded disk specimens were tested with a TA Instruments Discovery HR-2 rheometer (New Castle, DE, USA) using a 25 mm parallel-plate geometry and a gap of 2 mm. Specimens were initially heated to 200 °C using an environmental test chamber. Angular frequency was varied from 500 rad/s to 0.1 rad/s with a constant strain of 0.5%.

## 3. Results and Discussion

### 3.1. Observations from the Extrusion Process

During the extrusion process, there was a significant change in behavior when the CIP was used as the additive. The viscosity of the composite exiting the die was noticeably decreased, resulting in increased challenges in producing filaments with consistent diameters. To compensate for the changes in viscosity, the die temperature was reduced so that the exiting filament was solidified and consistent. These effects were not observed when the MS were included.

### 3.2. Density

The densities for PLA and the PLA composites are summarized in Table 1. The incorporation of microspheres at the 3.94 and 7.77 vol.% levels did not change the density appreciably. This was the result of the net change in the starting densities of PLA (ρ = 1.25 g/cm^3^) and MS (ρ = 2.2 g/cm^3^) being 0.95 g/cm^3^. The PLA composite samples with incorporated CIP exhibited much larger gains in density of 24.48% and 52.25% for 3.94 vol.% and 7.77 vol.%, respectively.

### 3.3. Thermal Behaviors and Degradation

**Figure 1:**
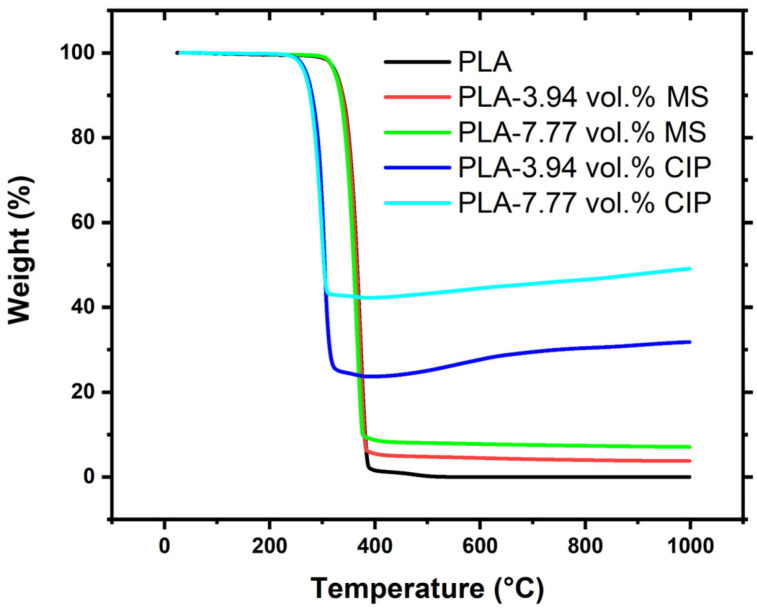
TGA data for PLA, PLA-MS, and PLA-CIP.

The effects of the additives on the thermal stability of PLA were investigated using TGA as shown in Figure 1. The neat PLA material had an onset degradation temperature of 349 °C. The microsphere-containing PLA composites at 3.94 and 7.77 vol.% exhibited similar degradation temperatures of 347 °C and 341 °C and had remaining weight % of 3.8% and 7.1%, respectively. The results for the PLA-CIP samples showed decreasing thermal stability where the onset degradation shifted to a lower temperature of 288 °C for 3.94 vol.% CIP and 278 °C for 7.77 vol.% CIP.

As the temperature was increased to greater than 400 °C, weight gain was observed in both CIP composites, indicating the oxide formation of the CIP from Fe to Fe_2_O_3_ [27,28]. At 400 °C, the weight remaining was 24% for 3.94 vol.% and 42% for 7.77 vol.%. By the end of the test at 1000 °C, the weight % remaining increased for both CIP samples to be 32% for the lower CIP-loaded sample and 49% for the higher CIP-loaded sample. The approximate change in the weight % for both PLA-CIP was 7–8 weight %. This change in temperature had a large impact on the processing conditions. The CIP had a higher density than the MS, and this impacted the weight % remaining even though the samples were processed at the same volume %.

Overall, the inclusion of the fillers changed the thermal stability of PLA. The MS particles did not greatly impact the onset temperature for degradation but produced a higher remaining weight % which corresponded to the loading of the MS into PLA. The addition of CIP caused the onset temperature of PLA to decrease by 61 °C for the 3.94 vol.% and 71 °C for the 7.77 vol.% loading. The metallic CIP created different heat transfer and degradation pathways than the neat PLA and ceramic MS particles.

**Figure 2:**
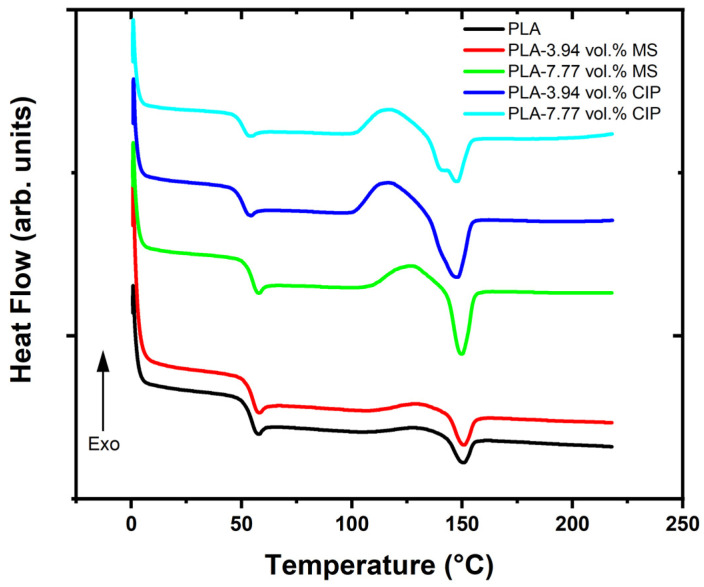
DSC data for PLA, PLA-MS, and PLA-CIP.

The DSC data are summarized in Figure 2 and Table 2. Shifts in T_g_ were observed and followed trends based on the additive type. The neat PLA control material exhibited a T_g_ of 53.0 °C. The insulating hollow MS produced similar T_g_ values of 53.8 °C for 3.94 vol.% MS and 53.5 °C for 7.77 vol.% MS, respectively. For the CIP-containing blends, the inverse relationship was observed with a decreasing T_g_. PLA-CIP had T_g_ values of 49.4 and 49.8 °C. The decrease in T_g_ followed similar trends reported on magnetic steel particles of a similar size range by Diaz-Garcia et al. [29]. The melting temperature (T_m_) characteristics among the samples did not follow the same trend. The composite samples all exhibited slightly lower T_m_ values compared to those of the neat PLA of 150.7 °C. The 3.94 vol.% MS sample had the lowest T_m_ of 144.2 °C. For the higher loading of CIP (7.77 vol.% CIP), two melting peaks were observed, and this has been shown in other PLA-filled systems to be due to the formation of more than one crystalline phase [16,29,30,31,32].

In all samples, a characteristic cold crystallinity (T_cc_) peak was observed. T_cc_ corresponded to the formation of crystalline phases that were not present at lower temperatures. For neat PLA and PLA-3.94 vol.% MS, the peak was very broad compared to the sharp peaks for the other composite samples. As the MS and CIP loadings were increased, the T_cc_ values decreased. For the MS-filled PLA, T_cc_ only decreased by 2 °C compared to the neat PLA, but for the CIP-filled samples, the T_cc_ values were reduced by 14 °C compared to the value of the neat PLA. The incorporation of fillers has been shown to impact the shape of the T_cc_ peak by making it more pronounced and sharper [29,31].

**Figure 3:**
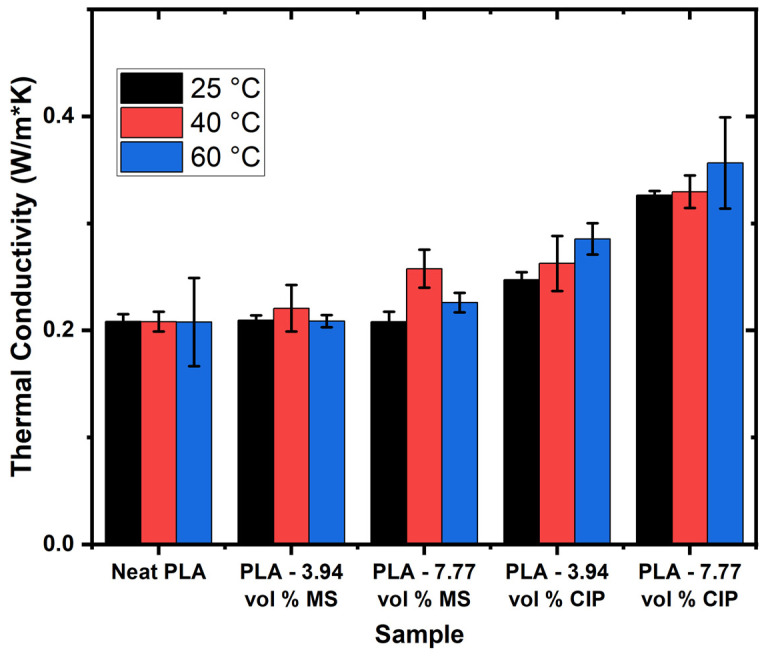
Thermal conductivity data at 25, 40, and 60 °C.

The thermal conductivity of PLA and the MS and CIP blends at room temperature (25 °C), 40 °C, and 60 °C is summarized in Figure 3. The room temperature behaviors of the samples were very similar to one another except for that of PLA-7.77 vol.% CIP. The PLA-7.77 vol.% CIP exhibited an almost 200% increase in the thermal conductivity when compared to the neat PLA. As the temperature was increased, the relationships between the MS and CIP and the neat PLA diverged. The CIP incorporation imparted an increase in the thermal conductivity, especially at higher temperatures. The reported CIP thermal conductivity value was 80 W/mK [33]. Laureto et al., 2017 observed similar behavior in PLA filaments with fillers composed of copper, bronze, magnetic iron, and stainless steel [12]. This corresponded to the need to lower processing temperatures using PLA-CIP composites since CIP can conduct heat so well. The inclusion of CIP at the two different loading levels created pathways suitable for increased thermal conductivity that were not present in the neat PLA or the PLA-MS composites. The thermal conductivity pathway is dependent on the type and amount of additive present [9,34,35]. These pathways can be isolated agglomerates, percolated networks, or conductive networks [33].

For the MS, the volume loadings were similar to those of the CIP, but since the MS was hollow, the thermal conductivity pathway and behavior may have traveled as if the matrix had large porosity. Xing et al., 2020 observed hollow glass spheres embedded in epoxy and described the decrease in thermal conductivity to be due to the air having a blocking effect on the heat transfer [36].

Another property of interest for the thermal properties of these PLA composites is effusivity or the ability for a material to exchange heat with another material when in contact [26]. This property can be correlated to the observation of PLA-CIP having a very low viscosity during the extrusion processing. The effusivity data trends as a function of particle material type and loading across the three different temperatures tested are shown in Table 3. The neat PLA exhibited the lowest values and the high-loading CIP (PLA-7.77 vol.% CIP) had the highest. Similar trends for particle loading increasing effusivity values have been observed in the literature for polysulfone composites of graphene and CNTs [37]. For PLA-based composites, the effusivity behaviors have shown a positive dependence on the infill rate and printing temperature [38]. This may be correlated to the decrease in thermal stability observed in TGA, as described previously.

**Figure 4:**
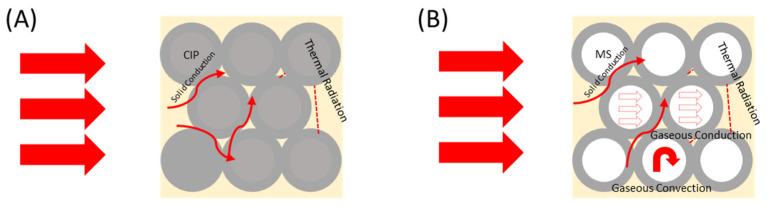
Illustration of thermal transport in (**A**) CIP and (**B**) MS particle packing.

As depicted in Figure 4, there are various pathways for heat transfer available through particles. In Figure 4A, the CIP is a solid filler; therefore, only solid conduction and thermal radiation are expected. For the hollow MS, Figure 4B visualizes the solid conduction and thermal radiation among the particles as well, but the empty void space within the particles can produce gaseous convection and conduction pathways that can produce an air blocking effect on the heat transfer [36].

### 3.4. Changes in Rheological Profile and Viscoelasticity

**Figure 5:**
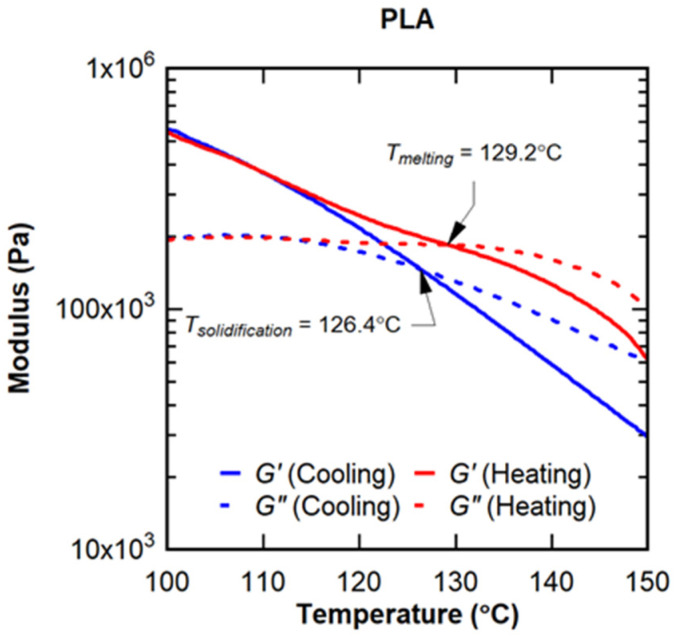
Example of viscoelastic crossover points in heating and cooling directions.

The data were analyzed to determine the temperature at which the storage (G′) and loss (G″) moduli were equivalent, which signified a fundamental change in behavior. When the storage modulus exceeded the loss modulus, the material behaved more elastically. When the loss modulus exceeded the storage modulus, the material behaved more viscously. The temperature when the two moduli were equivalent represented a phase transition. During the heating cycle, this temperature was indicative of when the composite realistically melted (T_melting_). During the cooling cycle, this transition temperature was indicative of when the composite solidified (T_solidifaction_). T_melting_ and T_solidification_ are important to understand for applications such as extrusion and additive manufacturing and allow for more energy-efficient protocols to be established. An example analysis is shown in Figure 5.

Table 4 shows the solidification and melting temperatures for the various combinations studied. Two things are revealed by these data. First, the addition of CIP to PLA reduced the necessary processing temperatures by more than 15% for both the heating and cooling cycles, regardless of the amount of CIP used. There were additional minor reductions in the processing temperatures for the high loading of CIP, but these were not as significant as those due to the addition of CIP to the neat PLA. The ceramic microspheres only influenced the heating cycle, with increases in the melting temperature observed for both loadings of microspheres. Interestingly, the lower loading of the microspheres had a greater effect on the melting temperature than the higher loading. This suggests some critical loading of the microspheres existed between the low and high dosages for the maximum increase in the melting temperature. Beyond this critical loading, the behavior of the continuous PLA phase began to dominate the behavior. Beyond the actual transition temperatures measured, it is also interesting to compare the differences between the solidification and melting temperatures for each composite. Little difference was observed for the neat PLA or for either loading of the CIP; however, differences of 24.5 °C and 13.4 °C were measured for the low and high loadings of the ceramic microspheres, respectively. For applications such as extrusion, this difference is critical. The processing temperature needs to be significantly greater than the die temperature for PLA composites with microspheres. The difference between the required processing and die temperatures for the neat PLA and composites with CIP is minor.

**Figure 6:**
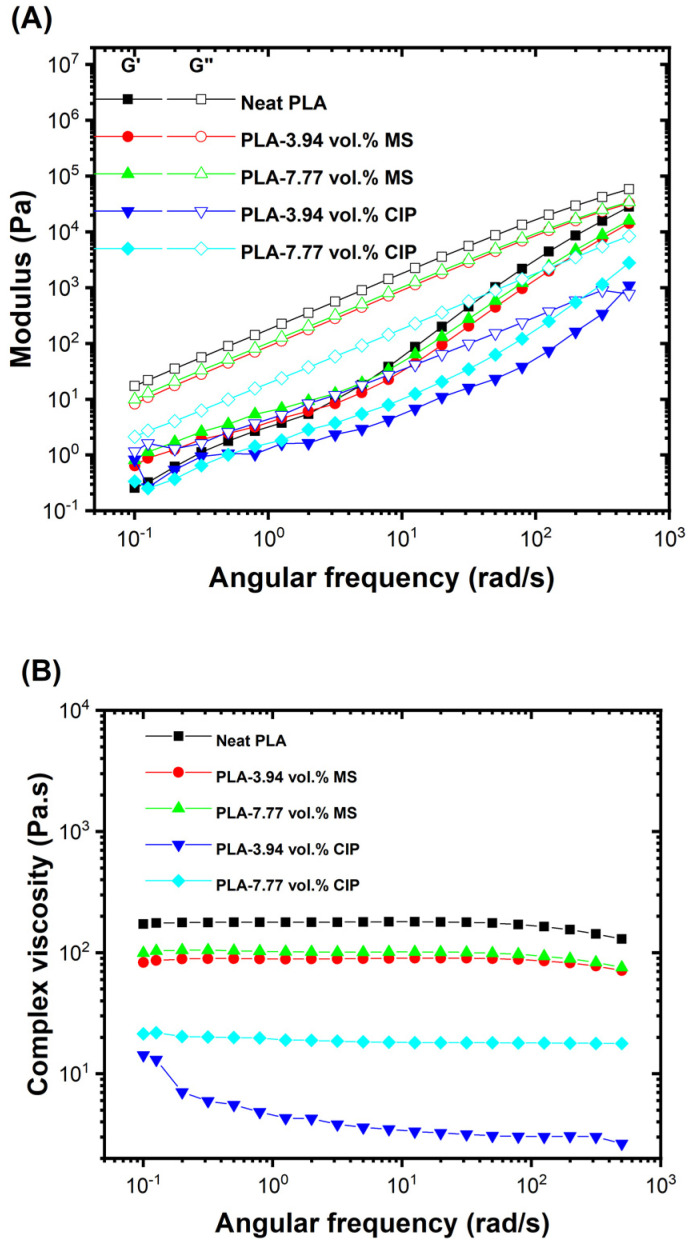
(**A**) Frequency sweep data and (**B**) complex viscosity data at 200 °C.

To further understand how the material behaved under temperatures simulating an extrusion processing environment, oscillatory frequency sweeps were conducted at 200 °C, as shown in Figure 6A. In most cases, G″ was greater than G′ which indicated liquid-like viscoelasticity behavior. For the 3.94 vol.% CIP, there was an observed G′/G″ crossover at 452 rad/s. The cause of the moduli crossover is not clear, but it could have arisen through the particle effects of the CIP. The magnitude of G″ followed a trend where the neat PLA had the highest G″ response which was then followed by the MS-based samples. The 3.94 and 7.77 vol.% MS samples were very similar in G″ and G′ behavior. The CIP-based composites exhibited lower G″ and G′ values compared to the neat PLA and PLA-MS, but the 3.94 vol.% CIP had a lower value for G″ compared to 7.77 vol.% CIP.

The complex viscosity behavior at 200 °C for PLA and PLA composites is shown in Figure 6B. Newtonian behaviors were observed for the neat PLA, PLA-MS composites, and the 7.77 vol.% CIP PLA. The 3.94 vol.% CIP sample had the lowest observed complex viscosity behavior but exhibited some slight shear thinning characteristics at lower frequencies. Compared to the neat PLA, the 3.94 vol.% CIP had more than an order of magnitude lower complex viscosity across all frequencies tested. This drastic drop in viscosity correlated with the observed behavior during the extrusion. The observed non-Newtonian behavior at this loading could be attributed to the overall low viscosity of the polymer melt and particle–particle interactions, causing potential viscous heating. This has been described as the “ball-bearing” effect [29]. The 7.77 vol.% CIP viscosity was also notably lower than that of the neat PLA and PLA-MS composites. The inclusion of CIP greatly affected the PLA rheology, but the effects of the particle–particle interactions started to produce an increase in the complex viscosity response once a high enough threshold of particle loading was reached.

## 4. Conclusions

The incorporation of functional fillers has a dramatic effect on the host polymer’s processing parameters and properties. PLA, as one of the most common thermoplastics used in FFF printing, has the potential to form a variety of composites. The inclusion of CIP at the loading levels studied here lowered the thermal properties of PLA, resulting in lower required processing temperatures. This was a direct effect of the density, specific heat capacity, and thermal conductivity. The CIP altered the thermal stability of PLA and reduced the onset degradation temperature by 71 °C at the highest loading of CIP. The viscoelasticity of PLA-CIP studied using frequency sweeps displayed almost an order of magnitude decrease in the complex viscosity at 200 °C, which is a common printing temperature of PLA. On the other hand, the inclusion of MS acted as a thermal insulator. The effects of the ceramic particles did not greatly affect PLA across many thermal properties. Interestingly, for PLA-MS, the G′/G″ crossover temperature point exhibited relatively large shifts, depending on if the composite material was being heated or cooled. The lower loading of MS (3.94 vol.%) had the highest observed crossover temperature of any material tested at 150.6 °C, which was nearly 25 °C different from the neat PLA. On the other hand, the CIP composites had the lowest crossover temperatures independent of heating or cooling and were about 20 °C lower than that of the neat PLA. Future work in this area will allow us to understand how the loading of functional fillers and the effect of percolation affects thermal properties, print quality, and mechanical performance.

## Figures and Tables

**Table 1:** Summary of density measurements and heat capacity values at 25, 40, and 60 °C.

Material	ρ (g/cm^3^)	C_p_ @ 25 °C (J/g °C)	C_p_ @ 40 °C (J/g °C)	C_p_ @ 60 °C (J/g °C)
PLA	1.242	0.602	0.663	0.563
PLA-3.94 vol.% MS	1.268	0.718	0.780	1.206
PLA-7.77 vol.% MS	1.295	0.657	0.704	0.977
PLA-3.94 vol.% CIP	1.546	0.392	0.438	0.793
PLA-7.77 vol.% CIP	1.891	0.316	0.361	0.562

**Table 2:** Thermal analysis data from DSC experiments.

Sample	Glass Transition Temperature (T_g_) °C	Melt Temperature (T_m_) (°C)	Cold Crystallinity (T_cc_) (°C)
PLA	53.0	150.7	130.8
PLA-3.94 vol.% MS	53.8	144.2	128.9
PLA-7.77 vol.% MS	53.5	149.8	126.8
PLA-3.94 vol.% CIP	49.4	147.7	116.9
PLA-7.77 vol.% CIP	49.8	147.5	116.8

**Table 3:** Effusivity data at 25, 40, and 60 °C.

Sample	Effusivity ((W × s^0.5^)/m^2^ × K))		
	25 °C	40 °C	60 °C
PLA	557.9	557.6	557.1
PLA-3.94 vol.% MS	559.0	571.9	558.1
PLA-7.77 vol.% MS	557.6	613.6	577.9
PLA-3.94 vol.% CIP	602.0	619.1	644.5
PLA-7.77 vol.% CIP	689.1	692.5	721.5

**Table 4:** Summary of G′, G″ crossover temperatures as observed during heating and cooling.

Material	$T_{solidification}$ (°C)	$T_{melting}$ (°C)	∆T (°C)
PLA	126.4	129.2	−2.8
PLA-3.94 vol.% MS	126.1	150.6	−24.5
PLA-7.77 vol.% MS	124.5	137.6	−13.4
PLA-3.94 vol.% CIP	107.6	107.3	0.4
PLA-7.77 vol.% CIP	103.7	102.0	1.7

## Data Availability

The original data presented in this study are included. Further data inquires can be requested from the corresponding author.
